# Ethical aspects of artificial intelligence: what urologists need to know

**DOI:** 10.1097/MOU.0000000000001278

**Published:** 2025-03-20

**Authors:** Rounak Verma, Findlay Macaskill, Anna Kim, Nicholas Raison, Prokar Dasgupta

**Affiliations:** a Responsible AI UK; b London School of Hygiene and Tropical Medicine; c Kings College London, London, UK

**Keywords:** algorithmic bias, artificial intelligence, artificial intelligence regulation, medical ethics, urology

## Abstract

**Purpose of review:**

The integration of artificial intelligence in urology presents both transformative opportunities and ethical dilemmas. As artificial intelligence driven tools become more prevalent in diagnostics, robotic-assisted surgeries, and patient monitoring, it is crucial for urologists to understand the ethical implications of these technologies. This review examines key ethical concerns surrounding artificial intelligence in urology, including bias, transparency, accountability, and data privacy.

**Recent findings:**

Recent literature highlights algorithmic bias as a significant challenge, where artificial intelligence models trained on nondiverse datasets may produce inequitable outcomes. The “black-box” nature of artificial intelligence systems complicates transparency and interpretability, raising concerns about clinician and patient trust. Emerging reporting standards, such as STREAM-URO and IDEAL frameworks, and WHO Guidelines provide structured approaches for ethical artificial intelligence integration in urology.

**Summary:**

The ethical deployment of artificial intelligence in urology requires a balanced approach that prioritizes fairness, accountability, and patient autonomy. Clinicians must advocate for explainable artificial intelligence, ensure equitable access, and integrate human oversight into artificial intelligence assisted decision-making. Future research should focus on improving dataset diversity, enhancing artificial intelligence interpretability, and establishing robust ethical guidelines to ensure that artificial intelligence advances align with medical ethics and patient-centered care.

## INTRODUCTION

Artificial intelligence in healthcare has a dual role, as both a diagnostic tool and a disruptor that reshapes the dynamics of doctor-patient interactions [1,2^▪▪^]. Key applications in urology can range from early detection of prostate cancer, robotic-assisted surgeries, and artificial intelligence driven wearable technologies for continuous and comprehensive monitoring of patients [2^▪▪^]. However, alongside these advancements, important ethical and professional challenges emerge, encouraging nuanced discussions on how urologists balance innovation with ethical responsibility. Historically, the adoption of newer technologies and medical breakthroughs, such as MRIs or robotic-assisted surgeries, has pressed for the need of robust frameworks and guidelines to establish fair and equitable use [3]. Artificial intelligence amplifies these dilemmas artificial intelligence the reliance on complex algorithms and large datasets that may obscure the decision-making processes, as well as introduce biases. In addition, when the health systems worldwide have extreme resource and infrastructure disparities, the ethical integration of artificial intelligence raises critical questions, especially around equity and inclusivity. Ensuring that technology does not reinforce existing inequalities is of paramount importance. This was the key focus of the World Bioethics Day 2024 [4] theme – “Nondiscrimination and Nonstigmatization” – which remains relevant in the discussion of all situations which pose a risk of exacerbating unequal opportunities.

This review discusses the core ethical aspects of AI adoption in urology by synthesizing literature, guidelines, and recent developments in the field. It is aimed to provide urologists with a solid foundation for comprehension and mitigation of the ethical challenges presented by artificial intelligence adoption. This further helps to ground the discussion within the larger framework of global health ethics and the rapidly changing landscape of artificial intelligence technologies. 

**Box 1:**
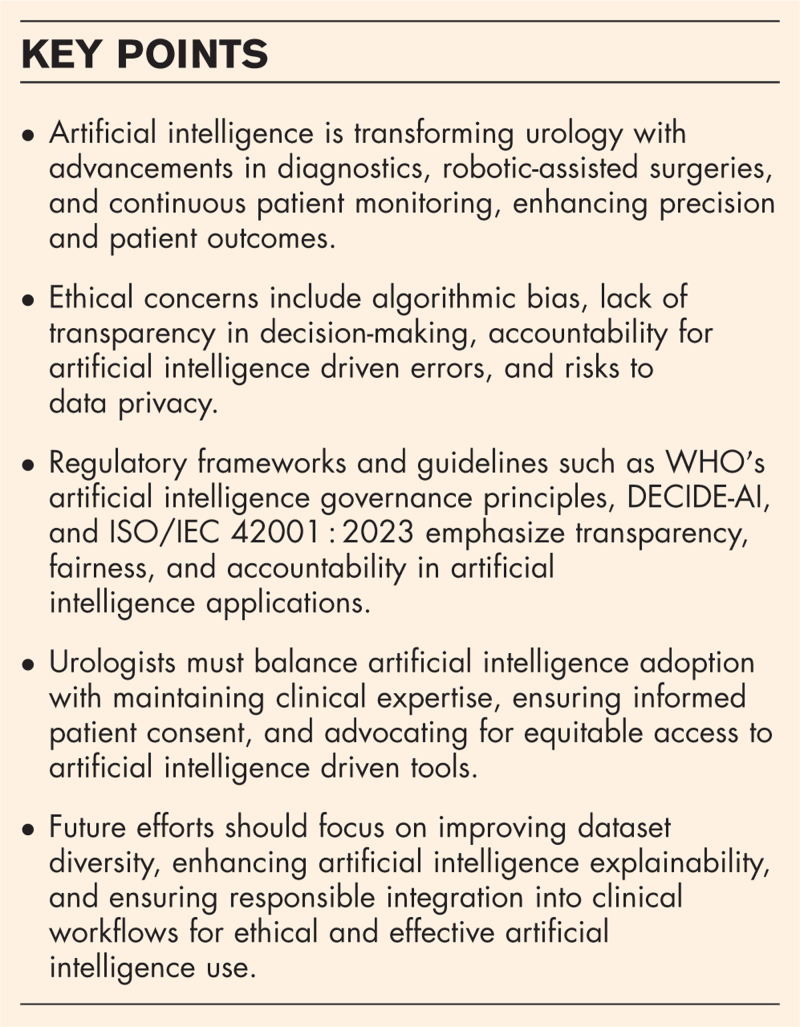
no caption available

## SCOPE OF ARTIFICIAL INTELLIGENCE IN UROLOGY

Artificial intelligence powered solutions are set to revolutionize the field of urology by facilitating accurate and reliable diagnosis of urological disorders ranging from prostate cancer to urinary tract infections. Algorithms trained on large imaging datasets have shown they can remove inter-observer variability by identifying subtle abnormalities that might escape the human eye, and facilitate early intervention and improvement in outcome [5].

Application of artificial intelligence powered robotic platforms like the da Vinci systems improve surgical accuracy, decrease complications, and shorten recovery periods [5]. Whether increased precision from robotic surgeries translates into measurable improvements in long-term patient outcomes remains a key ethical challenge that requires deliberation. Similarly, the economic implications of robotic systems – are they cost-effective or limited to high-resource settings – are also key factors that should drive adoption

Artificial intelligence enabled wearable devices allow for continuous monitoring of urological symptoms (e.g., urinary incontinence, nocturia). These tools provide clinicians with real-time data, while empowering patients to independently manage their own health. Moreover, artificial intelligence algorithms can be used to predict treatment responses, enabling more personalized therapeutic approaches [6^▪^]. But currently, these tools are primarily marketed across affluent socioeconomic groups.

Evidently as promising as artificial intelligence in urology is, it is not without its own challenges for implementation. One of the main issues is the poor generalizability of artificial intelligence models developed in high-resource environments [2^▪▪^]. Algorithms trained on noninclusive data fail to perform comparably in varied or low-resource settings [7^▪^]. Additionally, the complexity of artificial intelligence systems creates obstacles to adoption, as urologists must upskill and learn new technical skills to adjust to evolving technologies [1]. Solving these challenges will be vital to assuring that the advantages of artificial intelligence are accrued across the full range of urological care.

## ETHICAL CHALLENGES IN ARTIFICIAL INTELLIGENCE INTEGRATION

Use and integration of artificial intelligence tools in urology practice posits several ethical dilemmas, chief among them being the potential for algorithmic bias. As Cacciamani *et al.*[2^▪▪^] point out, the lack of diversity in training datasets often leads to disparities in artificial intelligence performance. For instance, prostate cancer detection models predominantly trained on data from white populations may yield less accurate results for patients from African or Asian backgrounds. This bias not only undermines the reliability of artificial intelligence tools but also exacerbates existing health inequities [7^▪^]. Addressing these issues requires concerted efforts to diversify datasets and incorporate demographic variables that reflect the broader patient population. Discussions must also emphasize on potential legal and reputational consequences for healthcare providers who use biased artificial intelligence tools for delivering care.

Another ethical concern is the “black box” nature of many artificial intelligence systems. These algorithms often operate in ways that are complex and not easily interpretable, even by the clinicians who use them, or the developers who conceptualize them. As Smith *et al.*[8] note, this opacity can erode trust between patients and providers. Explainable artificial intelligence, which focuses on creating models that are both accurate and interpretable, is therefore critical to maintaining trust, and enabling shared and informed decision-making.

Accountability is another pressing issue in the ethical integration of artificial intelligence. Collins *et al.*[3] highlight scenarios where errors in robotic surgery or artificial intelligence assisted diagnostics raise questions about who bears responsibility, the clinician, the developer, or the institution. Without clear guidelines for assigning accountability, the use of artificial intelligence in urology risks undermining both patient safety and professional integrity. Developing robust frameworks that delineate responsibilities and establish mechanisms for addressing errors will be essential to fostering accountability.

Data privacy and security are also critical ethical considerations. Artificial intelligence systems rely on vast amounts of patient data, raising concerns about confidentiality and the potential for misuse. The WHO 2024 [9] updated ethical guidance emphasizes the importance of implementing stringent data governance policies to protect sensitive information. In urology, where data often pertains to intimate aspects of patient health, breaches of confidentiality can have particularly severe consequences. The NHS Somerset AI [10] policy serves as a valuable model for how local governance, with small datasets where re-identification risks are higher, can prioritize data security while enabling the ethical use of artificial intelligence technologies. Complementing these efforts is the work of organizations like the Coalition for Health AI CHAI [11], which focuses on developing frameworks to ensure the ethical, equitable, and transparent implementation of artificial intelligence in healthcare.

Artificial intelligence adoption also raises critical ethical challenges related to climate change and sustainability. Training large-scale artificial intelligence models requires significant computational power, resulting in high energy consumption and carbon emissions [12]. The ethical dilemma lies in balancing the benefits of artificial intelligence-driven innovation with its carbon footprint, particularly in resource-limited settings.

## CURRENT FRAMEWORKS AND GUIDELINES FOR ETHICAL ARTIFICIAL INTELLIGENCE USE

Medical ethics are guided by four main principles: autonomy, beneficence, nonmaleficence, and justice [13]. Building upon this framework, Cacciamani *et al.*[2^▪▪^] have proposed a set of principles for artificial intelligence in urology that emphasize transparency, equity, accountability, and patient-centered care - to adequately address the unique challenges of artificial intelligence in urology-specific use cases.

Regulatory priorities around transparency, fairness, and accountability for artificial intelligence are reflected in recent legislations. In the US, the ACA Section 1557 Final Rule (June 2024) prohibits discrimination based on race, sex, or other protected attributes in medical artificial intelligence, whereas the HTI-1 Final Rule mandates transparency in medical decision support systems by requiring that the training and testing methodologies be disclosed. The UK AI Opportunities Action Plan describes strategic investment and regulatory frameworks to promote responsible artificial intelligence use while remaining compliant with ethical and legal requirements. Similar regulations such as the EU AI Act 2024 represent a global shift towards stringent testing, transparency, and accountability in the deployment of artificial intelligence [14].

Current reporting guidelines for artificial intelligence use include CONSORT-AI (Consolidated Standards of Reporting Trials – Artificial Intelligence), SPIRIT-AI (Standard Protocol Items: Recommendations for Interventional Trials – Artificial Intelligence), DECIDE-AI (Developmental and Exploratory Clinical Investigation of Decision-support systems driven by Artificial Intelligence), and TRIPOD-AI (Transparent Reporting of a Multivariable Prediction Model for Individual Prognosis or Diagnosis – Artificial Intelligence). These guidelines are designed to promote transparency, rigor, and reproducibility in artificial intelligence driven healthcare research [2^▪▪^].

Several guidelines for working in artificial intelligence are in development, addressing the unique challenges posed by medical artificial intelligence, as it continues to be used in new and innovative ways [2^▪▪^].

(1) STARD-AI (Standards for Reporting Diagnostic Accuracy Studies – Artificial Intelligence)
(2) PROBAST-AI (Prediction model Risk Of Bias Assessment Tool – Artificial Intelligence):
(3) PRISMA-AI (Preferred Reporting Items for Systematic Reviews and Meta-Analyses – Artificial Intelligence)

For artificial intelligence research specific to urology, the STREAM-URO (Standardized Reporting of Machine Learning Applications in Urology) [15] framework can be used to improve the transparency, reproducibility, and clinical relevance of artificial intelligence efforts in urology, as demonstrated by Khondker *et al.*[16] in a case example with pediatric hydronephrosis. Similarly, the IDEAL framework (Idea, Development, Exploration, Assessment, and Long-term Monitoring) [17] can provide a detailed, structured pathway for assessing new surgical technologies, with a thorough examination process ranging from assessing feasibility to off-the-shelf use in the real world.

ISO/IEC 42001 : 2023 [18] is a new standard that emphasizes the critical role of “ethics by design” in artificial intelligence development. For example, the standard outlines the need for clear documentation of algorithms, accessible explanations of artificial intelligence decision-making processes, and mechanisms to address unintended biases. By adopting ISO 42001 principles, urology practices can align their artificial intelligence applications with both ethical healthcare delivery and environmental sustainability.

In January 2024, the WHO released an updated version of their original 2021 [19] comprehensive Guidelines On Ethics And Governance Of Artificial Intelligence For Health. The aim of these Guidelines (2024) is to “assist Member States in mapping the benefits and challenges associated with use of Large Multimodal Models (LMM) for health and in developing policies and practices for appropriate development, provision and use”.

Responsible artificial intelligence UK [20] has also contributed to the development of ethical guidelines, advocating for interdisciplinary collaboration among clinicians, ethicists, policymakers, and developers. Such collaboration is essential to aligning artificial intelligence applications with societal values and clinical needs. Smith *et al.*[8] further recommend the creation of ethical training modules for clinicians, the formation of oversight committees to evaluate artificial intelligence tools, and the inclusion of patients in decision-making processes. These measures aim to foster trust and inclusivity while ensuring that artificial intelligence technologies are used responsibly.

## PRACTICAL IMPLICATIONS FOR UROLOGISTS

The integration of artificial intelligence tools in the near future creates a challenge for urologists to find a balance between harnessing the benefits of technological innovations while holding to the industry standards of ethics. Wiklund *et al.*[21] are concerned with over-reliance on artificial intelligence output, and discuss how they should feature in evidence-based decision-making. The goal here is to ensure the use of clinical judgment and utility of artificial intelligence as a valuable adjunct, thereby ensuring that clinicians do not lose critical skills with the advent of artificial intelligence, that is, deskilling. Transparency with patients also is essential. Clinicians must be very clear in their communications with patients about how artificial intelligence fits into their care, including why it may be beneficial – and how it may have limitations – in order to maintain informed consent and respect the autonomy of patients [22].

Generative artificial intelligence adds ethical concerns in academic and clinical training. While it enables personalized education and automated knowledge synthesis, it also risks misinformation, bias, and academic dishonesty. Over-reliance on artificial intelligence tools may undermine human expertise [23]. Artificial intelligence generated research articles and clinical guidelines may spread inaccuracies, as models cannot verify sources independently [24]. Studies warn of blurred lines between original scholarship and automated output, underscoring the need for clear policies on artificial intelligence disclosure and ethical use [25,26]. Institutions must enforce transparency, human oversight in verification, and ethical safeguards to prevent misuse in urology and medical academia.

Advocacy is another critical responsibility for urologists. Conveying open-ended questions, giving feedback on new artificial intelligence tools, and advocating for equitable access to these tools can help ensure patients have a say in how technologies are applied in their healthcare, ensuring fairness and accessibility throughout the process. Similarly, the concept of a “digital divide” within the urological profession itself opens avenues for discussion on how we ensure that all urologists, regardless of location, have access to training in artificial intelligence. The discourse at the 2025 World Economic Forum in Davos highlighted this need for equitable access, stating, “It is not acceptable that there are still people on our planet that don’t have access to healthcare with the vast amount of resources and technology available in the world” [27]. Urologists are ideally positioned to champion artificial intelligence solutions to reduce global inequalities, ensuring that new innovations benefit all patients regardless of geography or socioeconomic status.

## CHALLENGES AND FUTURE DIRECTIONS

Ethical governance of artificial intelligence in urology is still a work in progress. Traditional frameworks may be insufficiently granular, failing to address the nuances of robotic surgeries, diagnostic algorithms, and artificial intelligence powered training systems. Specific guidelines are required to ensure that these applications are aligned with underlying ethical principles whenever possible as well as appropriate to practical needs of clinicians and patients.

The speed of artificial intelligence innovation brings more challenges, such as how do we balance rapid technological progress with the need for robust oversight? Medical ethics must keep up with technology in order to be relevant and this will entail continued collaboration among clinicians, developers, and regulators. In addition, tensions exist amongst the bottleneck of resource inequality which makes equitable application of artificial intelligence tools even more difficult, especially for low-resource settings where access to advanced technologies may be thwarted. Tackling these inequities will demand new efforts to broaden access and tailor artificial intelligence systems to different healthcare contexts.

While current artificial intelligence systems in urology are highly effective for specific tasks such as imaging analysis, robotic surgery, and patient monitoring, the concept of artificial general intelligence (AGI) introduces new possibilities and risks for the field [28]. No fully autonomous surgical robots exist today, nor is it likely that such systems will be realized on any complex procedure soon Connor *et al.*[29,30]. But AGI, unlike narrow artificial intelligence, aims to emulate human-like reasoning, enabling it to adapt to diverse and unforeseen challenges. The promises of AGI are not without inherent risks and limitations, especially in high-stakes environments like healthcare [24].

Research from the Trustworthy Autonomous Systems (TAS) Hub [31] highlights the imperative role human oversight is expected to play in automated decision-making systems, particularly in high-risk domains such as surgery. The diversity of patient data available in the real world, the huge variability that exists when one actually treats a patient, calls for artificial intelligence applications in medicine to also have a “human-in-the-loop,” so that the application is well tolerated, adaptable in the real world, and also accountable. These datasets are particularly important because autonomous systems deployed in clinical settings would be subject to biases that could adversely affect patient populations; therefore, such systems are reliant on the expertise of clinicians, who would have constructive feedback on best practices for the systems’ deployment.

Future inquiry should focus on developing field-specific urology ethical frameworks that recognize the challenges unique to the specialty, much like the work undertaken by Cacciamani *et al.*[2^▪▪^,^32^]. Enhancing the diversity of training datasets is also crucial for minimizing bias and increasing the generalizability of artificial intelligence tools. Finally, investigating new approaches to increase the transparency of artificial intelligence systems and creating “ethical sandboxes” for testing artificial intelligence tools in controlled environments before broader adoption, may establish trust with clinicians and patients, leading to wider uptake of the technology.

## CONCLUSION

Artificial intelligence is projected as a disruptive technology with an unlimited potential to provide superior patient care and contribute to clinical practice advancement in urology. However, its application to healthcare needs to be guided by ethical principles — such as transparency, equity, accountability, and patient autonomy. Insights from Cacciamani *et al.*, Smith *et al.*, Collins *et al.*, ISO 42001 : 2023, and the WHO 2021, 2024, have provided frameworks that can help guide us through the ethical challenges of artificial intelligence in urology.

It will take active engagement from clinicians, policymakers, and developers to address these challenges. Through advocacy for ethical and transparent artificial intelligence, urologists can help ensure that technological advances uphold the very highest ethical standards in both delivering technological innovations and contributing to meaningful advances in patient care. And if integrated wisely, artificial intelligence can transform urology, paving the way for a future where technology aids, not undermines, the values of medical ethics.

## Acknowledgements

*The authors also acknowledge funding from the Trustworthy Autonomous Systems (TAS) Hub and UK Research and Innovation (UKRI). Additionally, they recognize support from the Wellcome Trust for Surgical and Interventional Engineering, the London Institute for Healthcare Engineering (LIHE), and King's College London (KCL).*

### Financial support and sponsorship

*This work was supported by the Engineering and Physical Sciences Research Council (EPSRC) [grant number EP/Y009800/1], through funding from Responsible AI UK (RAI UK). We also acknowledge funding from the Trustworthy Autonomous Systems (TAS) Hub and UK Research and Innovation (UKRI). We recognise support from the Wellcome Trust for Surgical and Interventional Engineering, the London Institute for Healthcare Engineering (LIHE), the Hinduja-King's Academy, Alberto Recordati, the King's-Vattikuti Institute, The Urology Foundation and King's College London (KCL).*

### Conflicts of interest

*There are no conflicts of interest.*

## REFERENCES AND RECOMMENDED READING

Papers of particular interest, published within the annual period of review, have been highlighted as:

▪ of special interest
▪▪ of outstanding interest
